# Analysis of Single Biomacromolecules and Viruses: Is It a Myth or Reality?

**DOI:** 10.3390/ijms24031877

**Published:** 2023-01-18

**Authors:** Tatyana O. Pleshakova, Yuri D. Ivanov, Anastasia A. Valueva, Victoria V. Shumyantseva, Ekaterina V. Ilgisonis, Elena A. Ponomarenko, Andrey V. Lisitsa, Vladimir P. Chekhonin, Alexander I. Archakov

**Affiliations:** Institute of Biomedical Chemistry, 10, Pogodinskaya St., 119121 Moscow, Russia

**Keywords:** reverse Avogadro’s number, single biomacromolecule, molecular sensors, atomic force microscopy, nanowire sensor, nanopore sensor

## Abstract

The beginning of the twenty-first century witnessed novel breakthrough research directions in the life sciences, such as genomics, transcriptomics, translatomics, proteomics, metabolomics, and bioinformatics. A newly developed single-molecule approach addresses the physical and chemical properties and the functional activity of single (individual) biomacromolecules and viral particles. Within the alternative approach, the combination of “single-molecule approaches” is opposed to “omics approaches”. This new approach is fundamentally unique in terms of its research object (a single biomacromolecule). Most studies are currently performed using postgenomic technologies that allow the properties of several hundreds of millions or even billions of biomacromolecules to be analyzed. This paper discusses the relevance and theoretical, methodological, and practical issues related to the development potential of a single-molecule approach using methods based on molecular detectors.

## 1. Introduction

Traditionally, biology and medicine have been recognized as the two major human life sciences. The overwhelming majority of studies are conducted within these sciences or at their intersection using bioanalytical methods, the sensitivity of which is based on the registration of a signal from millions or billions of objects [1]. Such studies do not allow one to understand the transformations of a single molecule over time, nor do they provide insights into the behavior of individual molecules with different conformations and properties. It is crucial to understand that populations of macromolecules that seem to be homogeneous and do not exhibit any chemical differences do have intermolecular variations (often referred to as “static clutter”) [2,3]. The results of such studies are the averaged characteristics of the properties of many objects, which are translated to biological systems.

In recent years, an entirely new field of science, often referred to as single-molecule biology or biochemistry, has emerged [4]. This alternative approach involves studying living systems at the level of individual molecules, with a focus on detecting the properties of single objects, such as biomacromolecules (nucleic acids, proteins) and viruses. This approach does not ignore the fact that viruses and biomacromolecules are the structures existing in the body as separate particles with characteristic physicochemical and functional properties.

An alternative approach at the level of single molecules suggests that the combination of “single-molecule approaches” will be opposed to “omics approaches”. From the practical point of view, developing a single-molecule approach, in which the object of study is a single molecule or virus, is strategically important because the emergence of biomacromolecules with significantly different properties is crucial for balancing the biosystem between the states of “health” and “disease”. The availability of methodological possibilities for identifying such molecules substantiates the fundamental possibility to switch biological and medical research from studying averaged properties (based on the analysis of complex molecular ensembles) to analyzing the properties of individual molecules.

This paper describes the starting points of single-molecule approaches in biology and medicine in comparison with omics approaches, outlines the limits of applicability of the panoramic and targeted approaches to the study of living systems, presents the existing methodological solutions for the detection of biomacromolecules, the determination of physicochemical properties, and functional features of individual molecules, and provides a computational rationale for the required level of analytical sensitivity for using the results in molecular biology and medical applications. The paper reports the results obtained using single-molecule approaches.

It should be noted that this review is not intended to provide any comprehensive analysis of all the methods and technologies discussed for detecting a single molecule and/or investigating its properties. The aim of this work is to present selected examples demonstrating conceptual advances and to highlight the comprehensiveness of approaches needed to develop improved methods for detecting and investigating individual properties of molecules.

## 2. Relevance: Properties (Phenomena, Relationships, and Effects) Determined for an Array of Objects Are Not Properties of a Single Object

An assessment of the applicability limits of omics (panoramic) and single-molecule (targeted) approaches to the study of living systems substantiates the need for new science.

Biosystems are characterized by a considerable diversity of individuals. The human population accounts for 8 × 10^9^ individuals, the number of prokaryotic cells ranges from 10^8^ to 10^12^, and the number of viruses in the universe possibly is as high as 10^31^ [5,6,7].

A typical genome differs from the reference human genome at 4.1 million to 5.0 million sites. Although >99.9% of variants comprise single nucleotide polymorphism (SNPs) and short indels, structural variants affect more bases, and the typical genome contains approximately 2100 to 2500 structural variants (~1.000 large deletions, ~160 copy-number variants, ~915 Alu insertions, ~128 L1 insertions, ~51 non-autonomous hominid specific retrotransposons (SVA) insertions, ~4 nuclear mitochondrial DNA segments (NUMTs), and ~10 inversions), affecting ~20 million bases of the sequence [8,9,10].

A typical genome contains 149–182 sites with protein-truncating variants, 10,000–12,000 sites with peptide-sequence-altering variants, and 459,000 to 565,000 with variants overlapping the known regulatory regions (untranslated regions (UTRs), promoters, insulators, enhancers, and transcription factor binding sites). Therefore, the number of nucleotides in the encodable genome portion different for two individuals (e.g., in the Russian population) would be ~(4.5 million × 1.15%) = ~52,000. However, not all of these are protein-altering variants. In healthy people, the percentage of genome differences amounts to 4.5 million/3.1 billion = 0.14%.

In the early twenty-first century, the human population was approximately 10 billion people, and in theory, could be almost infinite and comprise 102,000,000 individuals. Hence, humankind is represented by extremely diverse individuals. Personalized medicine is intended to take into account the specific features of biosystems at the level of the human organism and has been discussed by the global scientific community over the past decade [11,12,13].

A physician is the only specialist in the life sciences to deal with individual patients rather than an array of individuals (the human population). Other specialists (biologists and chemists) have conventionally studied objects as particle ensembles. Nuclear physicists investigating elementary particles are the only exception.

Therefore, a “unique” patient rather than an “average” member of a population comes to the physician. According to statistics [14], iatrogenic causes, including medical errors, are among the most common contributors to patient death. The diversity of individuals poses a specific concern for medicine because neglecting the individual characteristics of patients when making a diagnosis and choosing a therapeutic option can lead to mistakes.

In biology, there are actually examples of how a single molecule can affect the overall result. A single peptide antigen, for example, can activate a T-cell receptor and initiate an activation program that promotes an immune response [15]. One study [3] involved the investigation of mRNA expression in each cell, with a total of 50 cells analyzed. It was found that the average measured mRNA level was never consistent with the results obtained by individual measurements: there are “hard workers” performing very well, “lazy workers”, and cells not working at all (“saboteurs”). Among 50 cells, the measured mRNA levels in the cells never corresponded to the average value. However, the cellular function was preserved. Therefore, the study of systems at the single-molecule level would allow the objects determining the performance of the system to be identified. Therefore, studying single objects, including biomacromolecules and viruses, would provide new insights into the biosystem, which would allow creating a novel, “molecular” view of the world.

## 3. Methodological Solutions for Detection, Determination of Physicochemical Properties, and Functional Features of Individual Biomolecules

To assess the potential of studying single biomacromolecules, one should be aware of the resources that instrumentation engineering offers. The cutting-edge technologies required for novel science should offer the possibility to:

(a) Isolate single biomacromolecules (nucleic acids and proteins);
(b) Visualize and calculate macromolecules and viruses in a manner similar to how the Geiger sensor counting of beta particles (β) works;
(c) Manipulative individual macromolecules and viruses;
(d) Measure physicochemical properties and activity of single macromolecules and viruses.

It is important to solve the methodological problem of isolating the study object, i.e., a single molecule (nucleic acid or protein).

The polymerase chain reaction (PCR) underlying genomic analysis is critical for nucleic acid detection [16,17]. PCR is a molecular biology method that allows one to increase the number of nucleic acid fragments in a biomaterial by replicating single molecules. Currently, PCR is commonly used for sequencing and rarely for counting the copy number of nucleic acid molecules in biomaterials, mostly for diagnostic purposes. PCR-based medical diagnostics have been successfully used in modern clinical practice. For example, for COVID-19 detection, the information-carrying molecule within the virus is isolated, synthesized into a DNA string by reverse transcriptase, and then amplified by PCR until the number of molecules is large enough for a sensor to detect them. The number of PCR cycles is calculated based on the number of molecules to be detected.

Nanoengineering methods ensure that the study technique is independent of PCR efficiency. Individual DNA/RNA strands are passed through nano-sized orifices (biological nanopores) and used for analysis, with an electrical signal specific to each nucleotide within the biomacromolecule analyzed (in this case, the nucleic acid) being recorded. For the formation of a nanopore, membrane proteins are incorporated into the orifice of synthetic membranes, and an enclosed space capable of hosting a single biomacromolecule is formed. The membrane is subjected to voltage, and the ionic current flowing through the individual nanopores is recorded during the measurements. This signal can be used for sequencing nucleic acids, monitoring chemical reactions, and recognizing individual molecules. The situation is fundamentally different for protein analysis. With there being no “PCR” for protein molecules, an analog of the Geiger sensor (a device that records signals from a single molecule, i.e., a molecular detector) is needed.

The single biomolecule imaging approach tends to apply single fluorophore emission imaging techniques to study the movement and interaction of biomolecules and the interaction of biomolecules with cell components. Fluorophore studies are indispensable for investigating living cells and tissues, an important feature of which is the low contrast of internal structures. In summary, the main advantages of fluorescence microscopy are the possibilities to conduct intravital studies of objects and observe processes in real-time, to label specifically tissues, cells, organelles, and individual molecules, and to study several targets simultaneously with the currently available variety of fluorescent dyes and proteins [18]. However, all fluorescent microscopy methods share a common feature: the need for specific and accurate fluorescent labeling of biomacromolecules using biochemical and genetic methods developed for this purpose. The introduction of labels complicates the analysis procedure. It is debatable whether the data obtained using labeling can be attributed to real systems and whether the results of these studies can be applied to solve practical problems in medicine [19,20,21]. To sum up, all the methods in this group are “labeling” methods.

Methods that allow single molecules to be manipulated using perturbation of molecular systems on the nanometer scale, making it possible to study the energy of intermolecular interactions, the mechanical properties of biopolymers, and the kinetics of biochemical reactions [22]. The high sensitivity of force measurements using AFM, optical and magnetic tweezers provides an opportunity to determine intra- and intermolecular interactions at the level of single molecules. Thus, optical tweezers allow application forces up to 100 pN to particles, making them an ideal tool for mechanical impact on various biological objects and measuring their response. Over the past few decades, there have been numerous examples of successful applications of optical tweezers to investigate protein-nucleic acid interactions, protein/RNA folding, and molecular motors have been reported over the past few decades [23].

The last group includes the methods that can theoretically register the change of a specific physical parameter of a sensor element when interacting with a single biomolecule. For example, such methods include the nanoconductive detection method (NP-detector) and nanopore analysis.

In recent decades, the technology of nanowire biosensors has been intensively developed due to the fact that they are label-free detectors allowing macromolecules to be registered in real-time [24]. The NP biosensor operating principle is based on the registration of current modulation (or NP conductivity) flowing through the NP when analyte molecules are adsorbed on its surface. In such a biosensor, adsorbed molecules act as virtual gates, with NP having ohmic contacts at its ends acting as field-effect transistors (FETs). The high sensitivity of the NP-sensor element is due to the high surface/volume ratio [25]. As discussed in [26], the theoretical detection limit of a nanowire biosensor can reach the level of a single molecule per sensor element. So far, sensitivity at the level of single particles for NP biosensors has been achieved for viruses [25]. As for DNA and protein molecules, the experimentally obtained detection limit (DL) reaches the femtomolar level in the case of DNA [27], and even the subfemtomolar level in the case of proteins [28].

The nanopore-based method of analysis is a simple method for detecting single molecules. In contrast to other approaches for studying single biomolecules, including fluorescence-based methods, optical tweezers, and AFM, the analyte is not required to be fluorescent, labeled, or immobilized on a solid surface for biomolecule recognition [29]. Due to the simple electrical principle of registration, nanopores are probably the least complicated and cheapest method of detecting single molecules. A significant step in developing single-biomacromolecule science is the possibility to study the catalytic activity and kinetic parameters of individual enzymes (nanopore enzymology) [30,31,32]. Porins, outer membrane proteins, and toxins that form pores with a clearly defined space limited to hosting a single molecule are used as nanopores. Monitoring enzymatic reactions using nanopores has the unique advantage of long-term monitoring of native proteins at the single-molecule level and providing information on functional activity obtained from single molecules. So far, the concentration limit of protein detection with nanopore-based detectors is at least 10^−13^ M [33].

The uniqueness of the atomic force microscopy (AFM) method is that it can be related to all three groups of methods mentioned above. In other words, this method, which registers a signal from a single biomolecule, can be used both for imaging and manipulating molecules. The dimensions of the AFM sensing element probe are comparable to those of the protein molecules, making it theoretically possible to record the signal from a single protein molecule. The AFM sensing element interacts with a separate biological object located on the surface, and various characteristics of this interaction are recorded, allowing researchers to determine the physical properties of the object, such as its height, shape, and elasticity. The AFM visualization under conditions that allow for the reconstruction of biological processes, for example, complexation or enzyme cycles, makes it possible to relate physical properties to functional ones. There are many manufacturers of atomic force microscopes, including the Russian company NT-MDT (Zelenograd, Russia) offering a variety of measurement techniques to study biosystems under conditions similar to native ones. For example, AFM combined with fishing (a method of concentration) can be successfully employed for high-sensitivity bioanalysis [34,35]. Mass spectrometry analysis allows one to identify biomacromolecules in a system [36]. Pilot experiments have also been performed to measure the enzyme activity of single biomacromolecules using AFM [37,38,39].

## 4. Calculation Substantiations of the Required Level of Analytical Sensitivity for Using Results in the Practice of Molecular Biology and Medicine

The concept of “biomolecule concentration” requires a new interpretation when studying biosystems at the single-biomolecule level. This concept is routinely used to measure the concentrations of glucose, albumin, and bilirubin. The formula has been known for a long time, where concentration (*C*) is the normalized number of molecules.

$$C=\frac{Q}{V}$$

where *Q* is the number of macromolecules, mol; *V* is the volume.

In 2009, Ericsson [40] proposed to interpret the concept of “concentration” in terms of the intermolecular distance in the solution: unimolar—12 nm, millimolar—120 nm, and micromolar—1200 nm. Extrapolating this interpretation to the molecular level of the organism (particularly to blood, the representative biomaterial (1 μL) that is routinely used in bioanalysis for medical diagnostics), may allow the number of molecules of interest to be calculated. Table 1 lists the number of biomolecules per microliter of blood calculated for different concentrations.

Modern diagnostic medical systems are characterized by nanomolar levels of bioassay sensitivity [41]. Table 1 shows that a nanomolar (10^−9^) solution contains one billion molecules per microliter. One microliter of blood is the amount of biosamples to be collected from a patient during routine checkups or screening. Therefore, it is currently possible to detect pathological conditions involving more than one billion biomolecules.

As for detecting pathological processes, billions of molecules are already present in the advanced stages. Early diagnosis requires detecting single biomolecules that are pathology precursors and rare “witnesses” of deviations in the body [42]. For small tumors (<1 mm^3^ in size) [43] that cannot be visualized by modern imaging techniques such as magnetic resonance imaging, there are several millions of transformed cells, but it is unclear whether biomarkers are present in the blood. Hori and Gambhir [43] performed calculations using a mathematical model describing the kinetics of biomarkers in plasma with respect to tumor growth over time, starting with a single cancer cell. The calculations revealed that, for a tumor ~1 mm in diameter, the blood concentration of a single biomarker would be ~10^−15^ M. Therefore, it is difficult to detect pathology markers in the plasma because of their low concentration (~one marker molecule per microliter of blood). Rissin et al. [44] calculated that, for diagnosing cancer and viral diseases, the concentration detection limit of diagnostic methods should also be below 10^−15^ M.

Healthy individuals also have cancer cells, but their numbers are very low, with a blood concentration of 10^−18^ M (1 million cells) [45]. In precancerous conditions, the number of cancer cells is also low: 10^−18^–10^−13^ M (from 1 million to 1 billion cells). Patients with cancer have a larger number of such cells: >10^−13^ M (>1 billion cells). It is difficult to detect precancerous conditions because the available technologies cannot detect changes in the molecular profile of the body that can occur in cells as the pathology develops (i.e., the emergence of single biomarkers, even if they are known).

Table 1 shows that an attomolar sensitivity level of bioassay is essential: to assess the processes involving single biomacromolecules, a “Geiger sensor” for recording the signal from single biomolecules is required. Therefore, the attomolar level is the assay sensitivity range required for the novel scientific approaches.

Given a zeptomolar level (a 10^−21^ M solution), the recorded signal from a single molecule lies in the statistical probability range: only one molecule will be detected in one sample out of the collected 1000 samples. Using a “sensor” is unreasonable because it is statistically impossible to detect anything in 999 samples.

The estimated number of protein molecules in the blood could be ~2.2 million. However, in the best-case scenario, the data were obtained for about 2000 proteins [46]. The number of protein molecules in cells is small, with approximately half a million protein molecules per cell [47]. Let us determine the required analytical sensitivity for the following problem: detecting a single protein molecule in one cell.

The concept of Avogadro’s number in the context of analytical molecular biology was revised t the beginning of the twenty-first century [41]. Avogadro’s number has been widely used in biochemistry to calculate the molar characteristics of weakly concentrated solutions, where the number of molecules is significantly lower than that of solvent molecules. The transition to single biomolecules can be performed using the reciprocal of Avogadro’s *R_AV_* number, which can be written as:

*R_AV_* = 1/*N_A_* = 10^−24^ mole/molecules, or

*R_AV_* = 1 molecule/liter

So, seeking to calculate the number of copies of a protein (*N*) in a sample with a concentration (*C*) of 10^−18^ M in blood plasma, with a volume (*V*) of 1 μL, using the standard formulas for calculating the molar concentration, one would obtain one protein molecule in 1 μL. The reciprocal of Avogadro’s number is an important tool for calculating the number of molecules in specific biosystems. For example, the volume of plasma in the human body can be simplified as a volume of liquid of the order of five liters and the amount of protein can be determined using the traditional concept of “concentration”, but the such calculation is no longer suitable for a cell.

To evaluate the sensitivity of analytical systems, the concept of “reverse Avogadro’s number” was proposed. Accordingly, the sensor sensitivity significantly affects the sensitivity of the analytical system for protein detection. In the limiting case, when the sensor sensitivity was increased to a single molecule at an analyte solution volume of 1 L, the detection limit was 10^−24^, which is theoretically the normalized limit. It is convenient to use the reverse Avogadro’s number to estimate the number of biomacromolecules in a system for which it would be difficult to use the conventional concept of “concentration” (e.g., a cell).

When the blood was used for bioassays, it turned out that an attomolar sensitivity of analysis was required to solve the problem of detecting a single molecule in 1 μL of the sample, as shown earlier. This approach cannot be used for the cell because the concept of “concentration” is applicable to a specific volume of the analyte solution. When using the volume of a single cell for the calculations, one could solve the problem of detecting single molecules by analyzing 10^5^ cells. However, it should be mentioned that 10^6^ cells are used for proteomic analysis [48]. The analysis sensitivity depends on the cell volume: the smaller the cell, the higher the sensitivity of the method (Table 2).

Table 2 demonstrates that the analytical sensitivity required for monitoring molecular-level changes occurring in a single cell is much lower than that of modern technologies.

**Figure 1:**
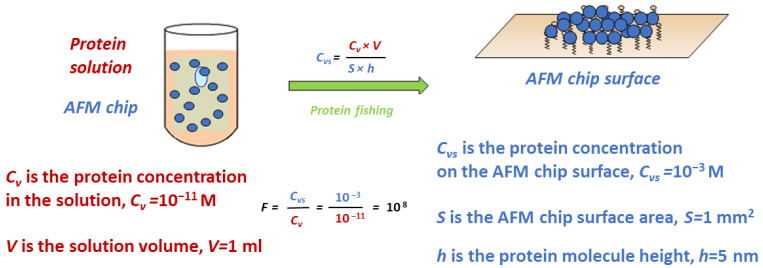
Schematic representation of the protein fishing process.

Let us focus on another aspect of interpreting the concept of “concentration” in the novel science. As mentioned above, AFM allows the signals from a single biomacromolecule to be detected. The necessary condition is the fixation of molecules on the surface (atomically smooth substrate). We have shown that chemical fishing can be used to force biomolecules to a specific zone (the so-called “delivery”) and immobilize them on the substrate. This type of fishing involves capturing biomolecules from a large volume of analyte solution to a small substrate surface and immobilizing them via covalent bonding between protein amino acid groups and active moieties on the surface (Figure 1). The covalent bonding contributes to capturing irreversibility and prevents the thermodynamically induced desorption of molecules from the surface. A concentrating efficiency of eight orders (concentrating factor of 10^8^) can be achieved by analyzing 1 mL of the solution [50]. Therefore, although the molecular concentration in the analyzed solution was 10^−11^ M, it was much higher on the surface (the near-surface volume occupied by molecules on the substrate): 10^−3^ M, which significantly facilitated molecule detection. This aspect of considering the concentration also makes it possible to integrate “single-molecule sensors” into the bioanalysis system.

## 5. Single Biomacromolecule Approaches: Examples of New Insights into Molecular Events

Below, we provide several examples demonstrating the potential of the novel technologies in single-biomacromolecule analysis: visualizing biomolecules and determining their oligomeric state, measuring the elastic properties of molecules, and evaluating functional activity. In general, the scope of the application of novel science overlaps with the problems solved using conventional biochemical methods.

Thus, the AFM visualization of the P450 protein adsorbed on the surface allowed the aggregate state of the protein in solution to be estimated: monomers (30%) and dimers (70%) have been detected [51,52]. Determining the aggregate state is a common biochemical problem. Protein solutions are used as drugs. The efficacy of the same drug, characterized by the same purity of protein material and excipients, may differ. This is quite plausible because the functional activity of a protein differs for different oligomeric forms, which are usually not studied.

**Figure 2:**
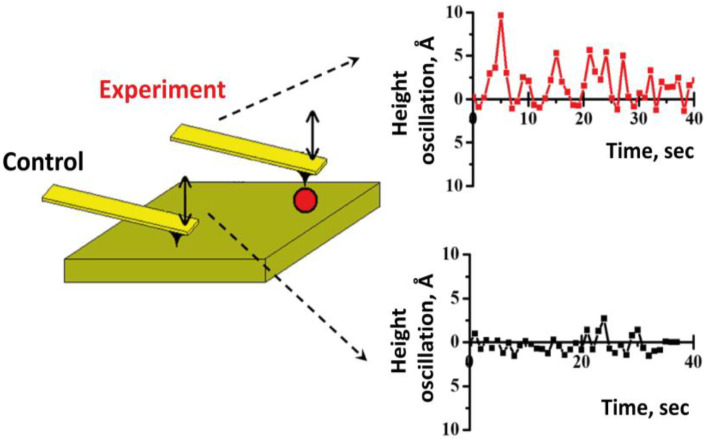
Monitoring the height of a single molecule during functional activity by AFM.

Pilot experiments aimed at assessing the activity of individual molecules using the AFM data showed the biomolecule height oscillation for the enzyme molecule (P450 CYP102A1) adsorbed on the surface (Figure 2). The oscillation amplitude depended on the stage of the functional cycle of the enzyme, allowing the functional activity (the measured changes in height expressed in angstrom (Å) units) to be estimated using the AFM data: the resting enzyme molecule, 0.60 Å, after adding a substrate (no activation, non-working system), 0.62 Å, after adding a second substrate (the system is activated), 1.43 Å, after adding an inhibitor (the system became inactive), 0.55 Å The data obtained showed that the signal from a single functionally active biomolecule was twice as high as that from a biomolecule in the inactive state. Comparable results were obtained using horseradish peroxidase. However, a pilot series of experiments revealed an interesting fact: monitoring 50 molecules demonstrated that there were “hard worker” and “lazy worker” molecules despite the active stage of the enzyme cycle. Therefore, the data obtained earlier [3] in the cell system by measuring mRNA expression were confirmed. The future areas of research will involve identifying a relationship between the AFM data (molecule height oscillation expressed in Å units) and protein activity and determining the parameters of the enzymatic reaction using classical biochemistry methods.

In medical diagnostics, novel AFM-based technologies have made it possible to visualize the results of successful complex formation between a novel molecular probe (aptamer) and the target protein (hepatitis C virus antigen). Oligonucleotide aptamers have advantages over molecular probes conventionally used in modern biosystems (antibodies) as platforms for medical diagnostic systems for biospecific interactions (e.g., ELISA). It has been demonstrated that the method can be used for visualizing and counting viral particles in a biological object (blood serum) [35]. Currently, AFM does not compete with ELISA and PCR for economic reasons. However, making the technology less expensive and upscaling it is a task for the future.

## 6. Conclusions

The life sciences have recently undergone revolutionary changes, similar to those that occurred in the 1980s and the 1990s. It is the creation of the PCR technology, which largely predetermined the advances in the Human Genome Project and was the most significant event in the late 20th and early 21st centuries and the basis for modern medical diagnostic methods.

An analogous situation has been observed over the past decade for a novel single-biomolecule approach. The first factor was inventing cryo-electron microscopy (cryo-EM). In 2017, the Nobel Prize was awarded for the development of cryo-EM to determine the structure of biomolecules in a solution with high resolution [53]. Cryo-EM is currently becoming a routine analytical technique [54,55].

In 2021, Google’s DeepMind team announced a breakthrough in using the AlphaFold 2 resource [56] to solve the fundamental research problem of predicting the 3D structure of any protein according to its amino acid sequence.

Combining cryo-EM technology and the AlphaFold 2 artificial intelligence program will allow novel drugs and vaccines to be designed at a fundamentally new level. The research journey between gene sequencing and the structure of a protein molecule synthesized by that gene will take only a few hours and cost tens or hundreds of USD, as opposed to months and hundreds of thousands or even millions of USD that were required before. In addition, combining novel technologies will allow making fantastic breakthroughs in designing new vaccines and medications. Some data indicating the efficiency of novel technology in this area have already been obtained. Coronavirus imaging data obtained with cryo-EM have been previously reported [57]. Furthermore, software-assisted processing has improved the resolution and detailed the viral spikes and glycosylated regions of proteins, validating the reasons for the absence of immunogenesis. It should be mentioned that the receptor-binding domain was discovered by V.N. Orekhovich in 1963 and was included in all the textbooks (ACE and ACE2 are the same and were discovered in Russia) [58].

Combining cryo-EM technology and the AlphaFold 2 artificial intelligence program is also essential for developing a novel single biomacromolecule approach. The strategy for studying biosystems at a new level is as follows:

1. Modeling the structure of a protein biomacromolecule;
2. Studying the biomacromolecule by cryo-EM;
3. Identifying properties and detecting the biomacromolecule using molecular sensors;
4. Identifying the role of each molecule in the biosystem.

The proposed strategy, applicable to a novel approach, can be used for designing vaccines. Fifty synthetic protein-based vaccines are currently undergoing various phases of FDA trials in the USA. This is a new approach that relies on synthetic vaccines whenever the appropriate circumstances arise, since these vaccines can be designed quickly. Synthetic vaccines can be designed using a carrier (nanoparticles or a protein) and a receptor binding to any domain that will be available for attack by the immune system (B-cells and T-cells) [59]. It is within the framework of the new approach that studying the structure and properties of a separate domain within a separate viral particle is effective because, as mentioned above, this will allow the role of each molecule in the biosystem to be identified. For example, the results of computer simulations of structural changes in a viral particle upon insertion or domain change can be verified using the cryo-EM method, and the preservation of affinity properties can be assessed based on AFM data. Designing a vaccine using the proposed strategy will significantly reduce the required resources (including time) compared with that of conventional methods.

To conclude, a novel single-molecule approach is a reality. Of course, there is still a need for novel technologies, original approaches, and new researchers thinking at the level of single molecules. However, developing and building an outlook for novel science is an urgent challenge for the scientific community. On the one hand, a new insight into the biosystem from the single-biomacromolecule perspective will allow one to form a new, “molecular” view of the world. On the other hand, given the necessity to accumulate a certain array of observations made by different research groups, interpreting the results of observations made in the novelty of science stills takes a long time.

## Figures and Tables

**Table 1:** Number of biomolecules in blood at different concentrations.

Concentration, M	Molecule Copy Number per 1 μL of Blood, Copies
10^−6^ (micromolar)	10^12^ (trillions of copies)
10^−9^ (nanomolar)	10^9^ (billions of copies)
10^−12^ (picomolar)	10^6^ (millions of copies)
10^−15^ (femtomolar)	10^3^ (hundreds of copies)
10^−18^ (attomolar)	1 (single copy)

**Table 2:** Assessment of analytical sensitivity sufficient for detecting a single molecule in the sample consisting of 10^6^ cells [49].

Type of Cells	Volume of Cells, L	Protein Concentration in the Cell, M	Sensitivity, M
Bacteria	5.0 × 10^−16^	10^−8^	10^−14^
Connective tissue cells *	6.3 × 10^−14^	2.7 × 10^−10^	10^−16^
Lymphocytes *	5.0 × 10^−13^	3.3 × 10^−11^	10^−17^
Hepatocytes *	4.0 × 10^−12^	4.0 × 10^−12^	10^−18^

* Data are reported for human cells.
